# Are Parents Getting it Right? A Survey of Parents’ Internet Use for Children’s Health Care Information

**DOI:** 10.2196/ijmr.3790

**Published:** 2015-06-22

**Authors:** Carolyne Pehora, Nisha Gajaria, Melyssa Stoute, Sonia Fracassa, Refilwe Serebale-O'Sullivan, Clyde T Matava

**Affiliations:** ^1^ The Hospital for Sick Children Department of Anesthesia and Pain Medicine Toronto, ON Canada; ^2^ The Hospital for Sick Children Surgical Day Care Unit Toronto, ON Canada; ^3^ The Hospital for Sick Children Department of Diagnostic Imaging Toronto, ON Canada; ^4^ University of Toronto Department of Anesthesia Toronto, ON Canada

**Keywords:** Internet, pediatrics, health information technology

## Abstract

**Background:**

The use of the Internet to search for medical and health-related information is increasing and associated with concerns around quality and safety.

**Objective:**

We investigated the current use and perceptions on reliable websites for children’s health information by parents.

**Methods:**

Following institutional ethics approval, we conducted a survey of parents/guardians of children presenting for day surgery. A 20-item survey instrument developed and tested by the investigators was administered.

**Results:**

Ninety-eight percent of respondents reported that they used the Internet to search for information about their child’s health. Many respondents reported beginning their search at public search engines (80%); less than 20% reported starting their search at university/hospital-based websites. Common conditions such as colds/flu, skin conditions and fever were the most frequently searched, and unique conditions directly affecting the child were second. Despite low usage levels of university/hospital-based websites for health information, the majority of respondents (74%) regarded these as providing safe, accurate, and reliable information. In contrast, only 24% of respondents regarded public search engines as providing safe and reliable information. Fifty percent of respondents reported that they cross-checked information found on the internet with a family physician.

**Conclusions:**

An unprecedented majority of parents and guardians are using the Internet for their child’s health information. Of concern is that parents and guardians are currently not using reliable and safe sources of information. Health care providers should begin to focus on improving access to safe, accurate, and reliable information through various modalities including education, designing for multiplatform, and better search engine optimization.

## Introduction

The use of the Internet to search for medical and health-related information is increasing in Canada and worldwide [1-4]. Of major concern regarding the use of the Internet for health-related issues is the quality of information found there. The information can be poor, and users may not be able to assess the quality and reliability of websites they are using [5]. Previous studies have shown that parents are using the Internet to search for health information for their children [4-9]. Parents’ perceptions of the quality, reliability, and usefulness of websites vary [5-10]. We undertook a survey to identify the habits of parents and guardians using the Internet to search for children’s health information and to determine their knowledge of safe and reliable sites. The aim of this study was to understand the current use and patterns of access to reliable and accurate Internet-based health information by parents and guardians of children seen at our institution.

## Methods

### Ethics and Setting

Institutional approval was obtained before starting the survey, participants were informed that consent was implied by participation in the study.

Following a pilot among parents in January and February 2013, the survey took place between May and July 2013 in the parent waiting room of the Hospital for Sick Children, a pediatric tertiary care facility in Toronto, Canada. Surveys were conducted after the children had gone into the operating room.

### Survey Design

A review of the literature discussing websites used by parents informed the development of the survey instrument. A 20-item survey instrument was constructed using the online tool, Qualtrics Offline (Qualtrics, LLC), based on previously published methodology for the design of surveys [11]. After pretesting was performed by the authors and a pilot was tested on parents, the final survey tool was loaded onto a tablet computer for administering to parents and guardians.

The final survey tool (Multimedia Appendix 1) elicited information on current patterns of Internet use by parents searching for information on their child’s health, types of devices used to access information, perceived reliability of websites and cross-corroboration of information, and future uses of the Internet for child’s health information.

### Sample Size Survey Distribution and Collection

With 16,000 surgical procedures performed in our institution annually, assuming a 95% confidence interval and an 8% margin of error, 149 participants were required. Our target population and sampling frame consisted of parents and guardians of children presenting to the preoperative waiting room for same day surgery and procedures. Excluded were parents who did not speak/understand English, those who declined to participate in the survey, and those who were employees at the hospital.

Three researchers conducted the survey and entered responses onto a tablet computer in real time; responses were then transferred to an Excel spreadsheet (Microsoft Corporation) for analysis.

### Data Analysis

Data analysis was performed using Prism version 5.0b for Mac OS X (GraphPad Software); descriptive statistics were used to summarize the data.

## Results

### Demographics

Of the 146 parents agreeing to participate in the study, 125 (85.6%) spoke English as their first language (Table 1). Most parents (108, 74.0%) reported that their child had been previously admitted to the hospital or attended an emergency/walk-in clinic.

**Table 1:** Primary language of respondents.

Primary Language	n (%)
English	125 (85.6)
Spanish	5 (3.4)
Farsi	3 (2.1)
Arabic	2 (1.4)
Gujarati	2 (1.4)
Albanian	1 (0.7)
Bulgarian	1 (0.7)
Chinese	1 (0.7)
Hindi	1 (0.7)
Hungarian	1 (0.7)
Persian	1 (0.7)
Somali	1 (0.7)
Twi	1 (0.7)

### Current Use of the Internet

**Figure 1:**
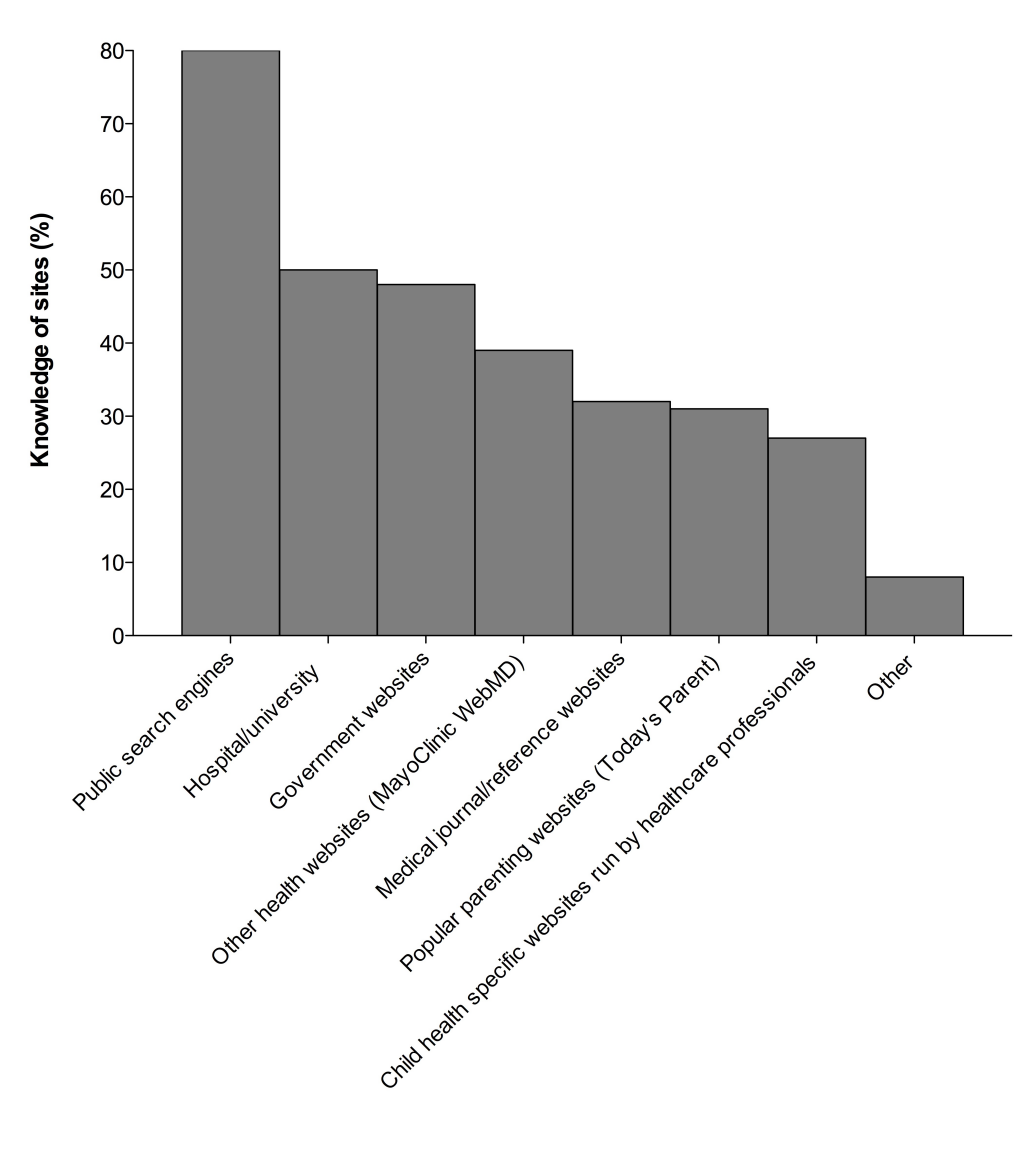
Respondents’ familiarity with various websites for access to information on children’s health.

**Figure 2:**
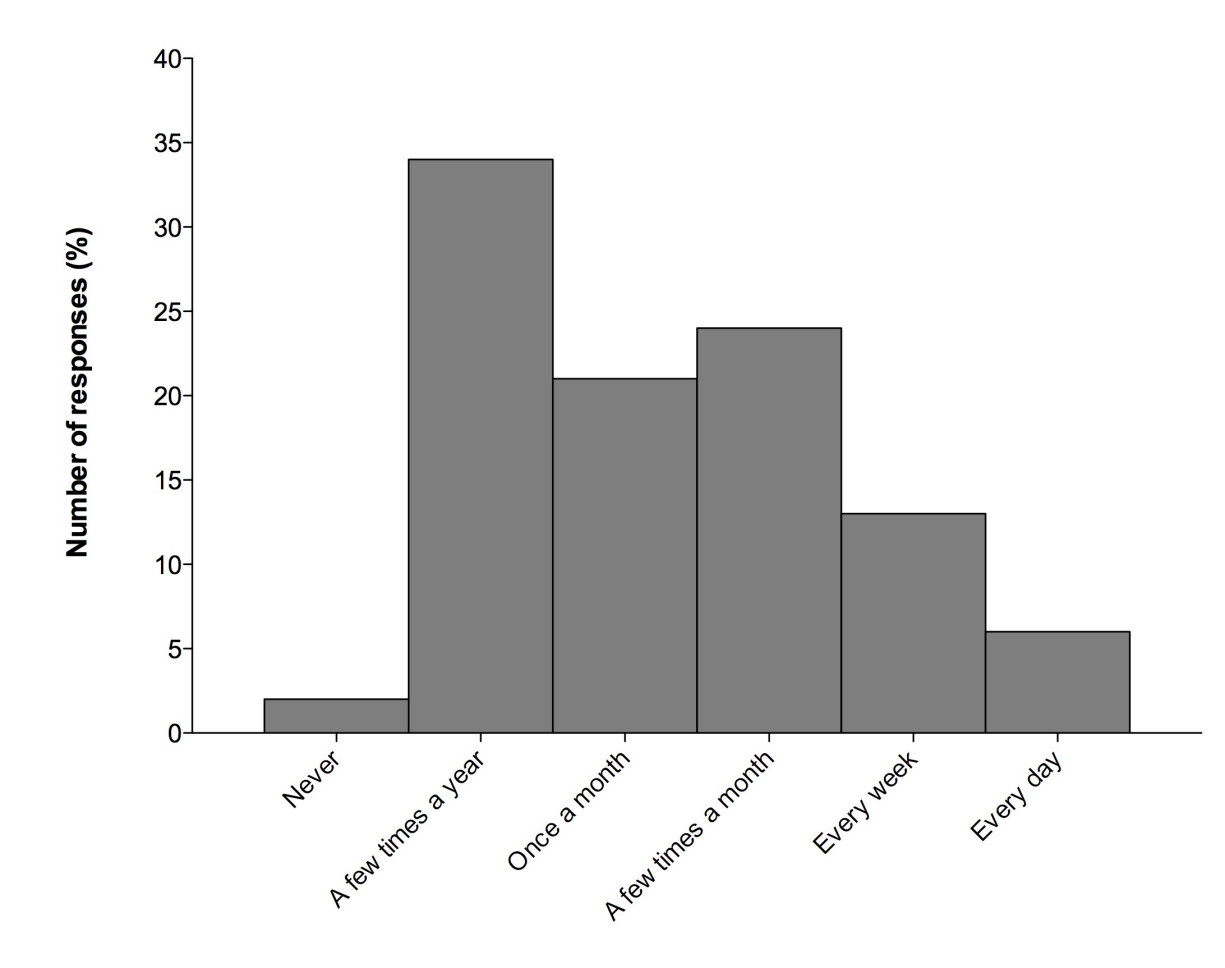
Frequency of Internet use for health related information.

Almost all parents (143, 97.9%) reported using the Internet to search for health information regarding their child. Parents reported being most familiar with public search engines as a source of health information (117, 80.1%) and least familiar with child-specific websites run by healthcare professionals (39, 26.7%) (Figure 1). Forty-three percent of respondents reported that they searched for health information regarding their child frequently, between a few times a month to every day (Figure 2). Parents reported using laptop computers, desktop computers, mobile phones, and tablets in almost equal proportions (90, 61.6%; 77, 52.7%; 67, 45.9%; and 63, 43.2%, respectively). Only 34.9% of respondents (51) reported that they knew our facility had a website dedicated to information on children’s health.

### Characterizing Use of the Internet

**Figure 3:**
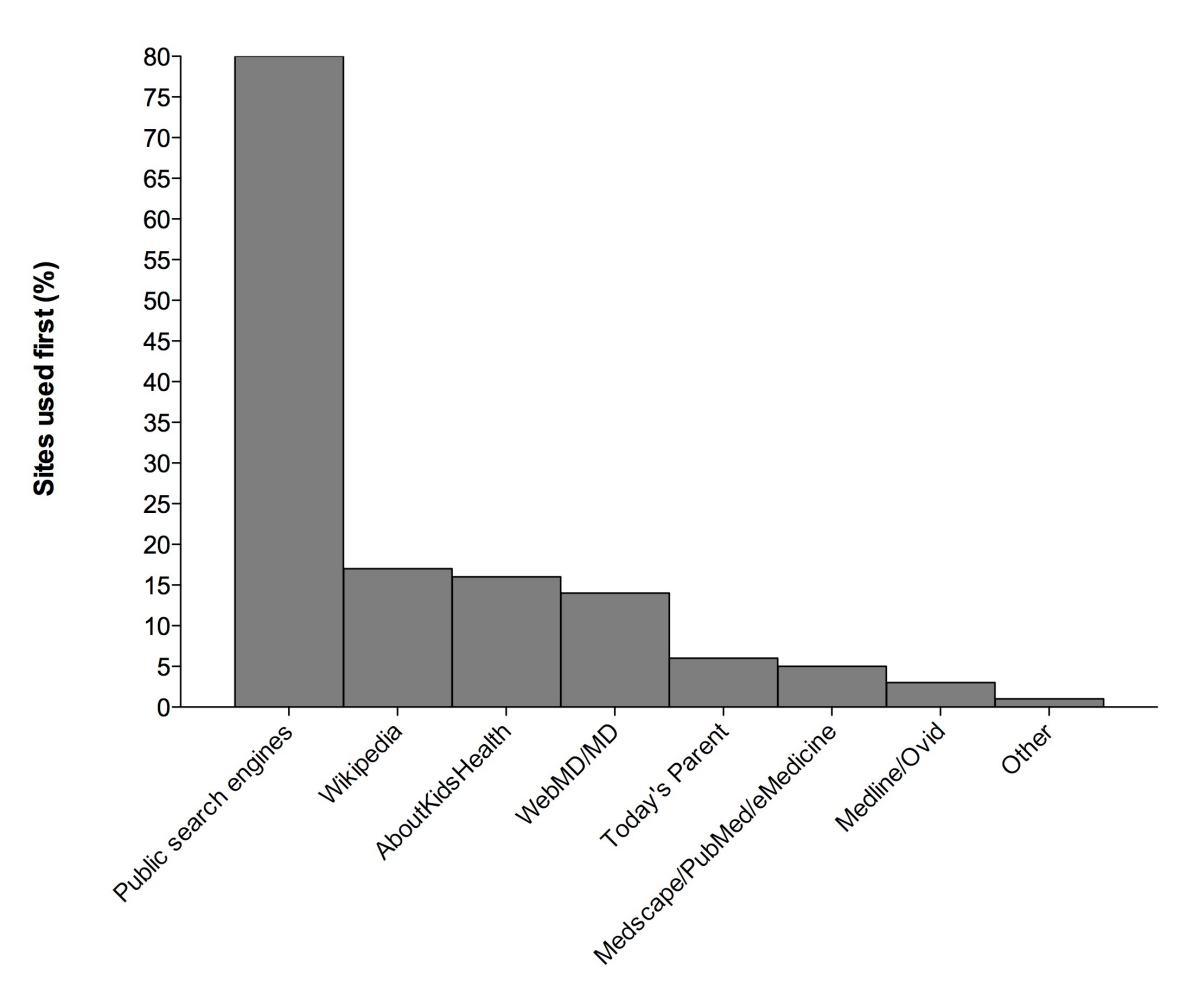
Sites first accessed for health-related information.

Public search engines were used as a starting point by 117 respondents (80.1%) (Figure 3). The majority of parents (126, 86.3%) reported searching for information on common conditions such as colds/flu, skin conditions, and fever; only 23 (15.8%) reported seeking information about surgical/diagnostic procedures that were related to the child’s presentation on the day of the study. A complete list of child health issues respondents reported searching about and reasons associated with the search is included in Table 2.

**Table 2:** Information sought and factors influencing Internet searching.

Search Topics		n (%)
**General information**		
	Specific conditions	126 (86.3)
	Surgical/diagnostic procedures	23 (15.8)
	Child development	15 (10.3)
	Diet/feeding	7 (4.8)
	Medications/treatments	4 (2.7)
	Child behavior	3 (2.1)
**Specific conditions**		
	Cough, cold, flu	22 (15.1)
	Skin conditions (rash, eczema, acne)	22 (15.1)
	Fever	19 (13.0)
	Cancer	16 (10.9)
	Allergies	13 (8.9)
	Congenital heart disease	9 (6.2)
	Asthma	9 (6.2)
	Eye conditions (strabismus, cataracts)	9 (6.2)
	Abscesses, cysts	9 (6.2)
	Gastrointestinal (vomiting, diarrhea)	9 (6.2)
	Hypospadias	7 (4.8)
	Chromosomal abnormalities	7 (4.8)
	Diabetes	6 (4.1)
	Cleft lip/palate	6 (4.1)
	Bone lesions	6 (4.1)
**Reasons for searching**		
	Health concern/question	99 (67.8)
	Upcoming surgery/procedure	7 (4.8)
	New diagnosis	4 (2.7)
	New medication/treatment	4 (2.7)
	Need for additional information not provided by health care practitioner	4 (2.7)
	Urgent need for information	4 (2.7)
	Availability of doctor	4 (2.7)

### Perceptions on Reliability of Websites and Information

**Figure 4:**
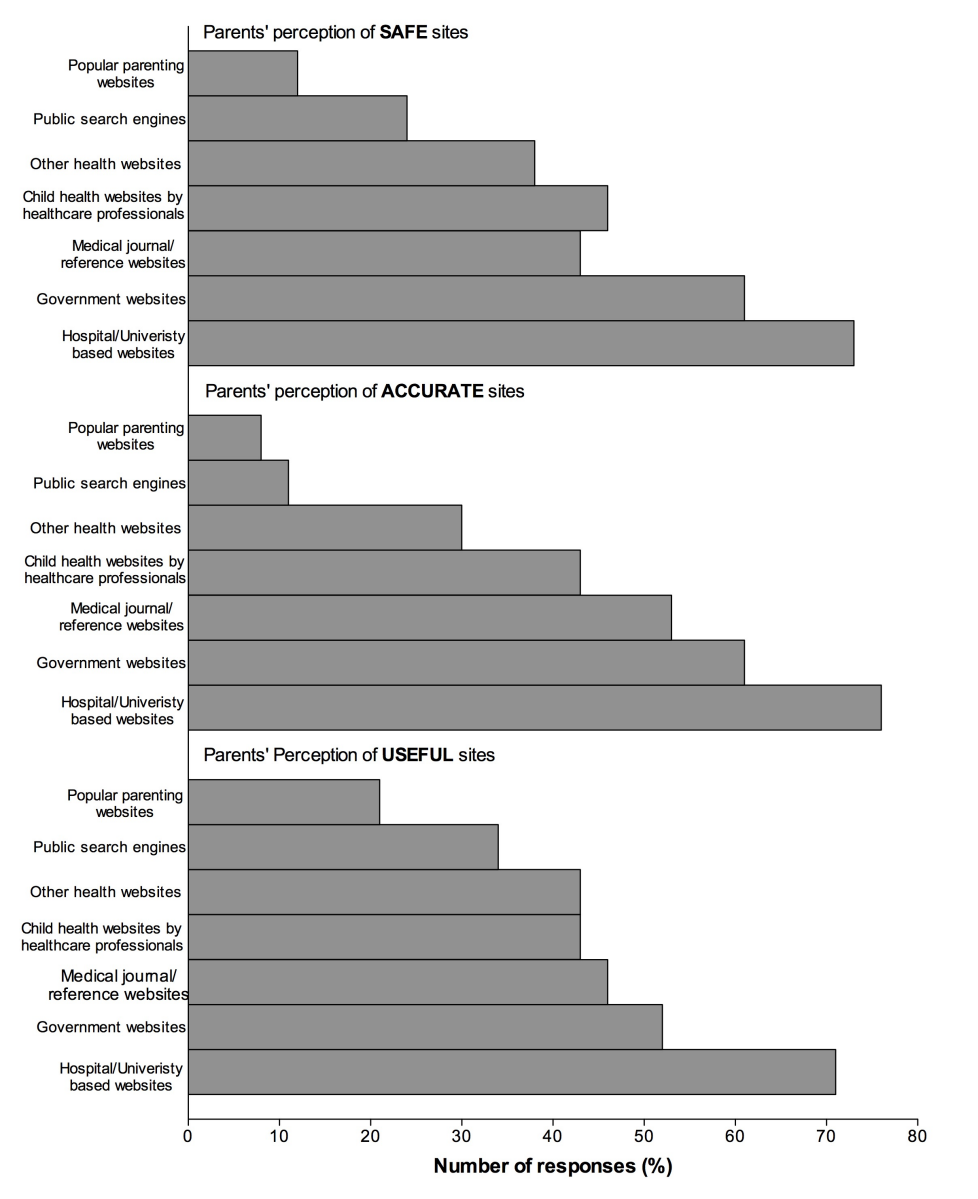
Parent’s perception of safe, accurate and useful websites.

Parents reported that they regarded hospital/university websites as safe, accurate, and useful sources of health-related information (105, 71.9%; 104, 71.2%; and 111, 76.0%, respectively). Very few parents regarded public search engines and popular parenting websites as safe sources (35, 23.9% and 18, 12.3%, respectively) or accurate sources (16, 11.0% and 12, 8.2%, respectively)(Figure 4). Fifty-one percent of parents (74) reported that they crosschecked health-related information found on the Internet with a family physician, with a pediatrician (67, 45.9%), family (64, 43.8%), friend/family in healthcare (54, 37.0%) and friends (45, 30.8%). Regarding future use of the Internet (in the next 12 months) for child health information, 112 (76.7%) of parents reported that they were likely to use our facility’s website following this study and 82 (56.1%) reported that they will continue to access and cross-check information found through public search engines (Table 3).

**Table 3:** Websites parents were likely to use in the future for their child health information and crosscheck information they found.

Primary sources, n (%)				
	Not likely	Neutral	Likely	No answer
Hospital/university-based websites	44 (30.1)	15 (10.3)	83 (56.8)	4 (2.7)
Medical journal/ reference websites	64 (43.8)	22 (15.1)	56 (34.8)	4 (2.7)
Government websites	36 (24.7)	19 (13.0)	84 (57.5)	7 (4.8)
Public search engines	16 (11.0)	19 (13.0)	107 (73.3)	4 (27.3)
Other health websites	61 (41.8)	10 (6.8)	65 (44.5)	10 (6.8)
Popular parenting websites	82 (56.2)	14 (9.6)	39 (26.7)	11 (7.5)
Likely to cross-check information from the following websites, n (%)				
Hospital/university-based websites	33 (22.6)	14 (9.6)	93 (63.7)	6 (4.1)
Medical journal/ reference websites	38 (26.0)	21 (14.4)	81 (55.5)	6 (4.1)
Government websites	36 (24.7)	19 (13.0)	85 (48.2)	6 (4.1)
Public search engines	16 (11.0)	19 (13.0)	107 (73.3)	6 (4.1)
Other health websites	40 (27.7)	15 (10.3)	81 (55.5)	10 (6.8)
Popular parenting websites	39 (26.7)	14 (9.6)	81 (55.8)	12 (8.2)

## Discussion

### Principal Findings

The results of our study demonstrate that almost all respondents use the Internet to search for child health information. Of the 97.9% (143) of parents and guardians who use the Internet to search for information, almost half were frequent users who searched anywhere from a few times a month to every day. This represents a significant increase in the use of the Internet compared to previous studies [4,6-9]. The use of the Internet by parents of patients in pediatric tertiary care centers in Australia, Canada, and the United States rose from 40% to 71% between 2003 and 2006 [4,6-9]. The continued rise reflected in our findings may be explained in part by the proliferation of high speed Internet in homes and on mobile devices. In addition, parents are increasingly taking an active role in their children’s health care and using the Internet to seek information around their child’s health [6]. The high level of use reported in our study may demonstrate the need for health care institutions and governments to continue to invest in the provision of online health care information about children for parents and guardians.

While the respondents participating in our study were at a tertiary care facility, the majority sought information on common childhood ailments (cold/flu, skin conditions, and fever) with a surprisingly smaller proportion (23, 15.7%) searching for information about an upcoming surgery or procedure. The development of web content pertaining to common conditions may remain a key focus for health care providers, web content developers and public health officers.

The majority of respondents reported that they use public search engines to search for health-related information for their child. Despite the choice of public search engines to search for health-related information, respondents rated public search engines low in terms of providing access to safe and accurate information. The use of the public search engines to search for health-related information raises concerns as several authors have reported the quality of health information found on the Internet to be poor [5,12-15]. Public search engine results provide links to various information sources that may result in misinformation, information overload, mistrust in the health care system by the parents and guardians, and potential detrimental effects on a child’s health. Respondents in our study identified government, university, and hospital-based websites as sources of safe, accurate, and reliable information; however, they also reported being less likely to access these websites for health-related information. A possible reason is that parents may not know of the existence of government, university, and hospital-based websites that would provide such valuable, safe, accurate, and reliable information. Previous studies have reported that parents and guardians would appreciate some guidance about searching for information on the Internet [4]. Health care providers can meet that need by providing lists of reliable websites through print, email newsletters, television, and radio drives [15]. Search engine optimization of websites can increase their visibility in search results, important because anecdotal evidence suggests that users do not navigate past the first page of results.

Our study demonstrates that almost half of respondents reported that they cross-checked information found online with a family physician. Reasons for this may include a desire to check the reliability and accuracy of information or get clarification. This cross-check could potentially be linked to a telehealth presence, chat line, or mobile apps to help parents who have questions or concerns about what they have read about on the Internet.

### Future Use of the Internet by Parents

Respondents in our study reported they were likely to continue to use desktop computers, laptops, tablet devices, and smart phones in almost equal proportions to search for information on their children’s health. Health information providers should consider designing websites that can be viewed and navigated easily on multiple platforms.

### Limitations

We used a convenience sample of parents of children presenting for surgery at a tertiary care hospital, so it is possible that the results may not reflect the general population. It would be important, when developing websites for individual facilities, that a needs assessment be done to ensure that the information contained on the website meets the needs of the intended audience.

We excluded parents who did not read and understand English. The patterns of Internet use may be influenced by culture and language, something our study did not investigate.

Finally, we relied on parents to self-report sources they access, which may be associated with recall bias. Our study had a large sample size and adequate response rate providing meaningful data for analyses and drawing significant findings and conclusions.

### Conclusions

Our study demonstrates that almost all parents are using the Internet to seek health care information related to their children. Information sought is largely related to general childhood health issues and development. Parents do not routinely use websites that are known to provide safe, accurate, and reliable information. Health care providers should focus on improving access to safe, accurate, and reliable information for parents and guardians. The development by policy makers of resources to educate parents and guardians on how to use the Internet to search for health information may aid in improving this area of medicine.

## Appendix

Multimedia Appendix 1 Parent survey.

## References

[1] Statistics Canada.

[2] Office for National Statistics.

[3] Fox S, Duggan M (2013). Health online.

[4] Khoo K, Bolt P, Babl FE, Jury S, Goldman RD (2008). Health information seeking by parents in the Internet age. J Paediatr Child Health.

[5] Wainstein BK, Sterling-Levis K, Baker SA, Taitz J, Brydon M (2006). Use of the Internet by parents of paediatric patients. J Paediatr Child Health.

[6] Semere W, Karamanoukian H, Levitt M, Edwards T, Murero M, D’Amcona G, Donias H, Click P (2003). A pediatric surgery study: Parent usage of the Internet for medical information. J Pediatr Surgery.

[7] Dhillon AS, Albersheim SG, Alsaad S, Pargass NS, Zupancic JAF (2003). Internet use and perceptions of information reliability by parents in a neonatal intensive care unit. J Perinatol.

[8] DeLuca JM, Kearney MH, Norton SA, Arnold GL (2012). Internet use by parents of infants with positive newborn screens. J Inherit Metab Dis.

[9] Naftel RP, Safiano NA, Falola MI, Shannon CN, Wellons JC, Johnston JM (2013). Technology preferences among caregivers of children with hydrocephalus. J Neurosurg Pediatr.

[10] Sim NZ, Kitteringham L, Spitz L, Pierro A, Kiely E, Drake D, Curry J (2007). Information on the World Wide Web: how useful is it for parents?. J Pediatr Surg.

[11] Passmore C, Dobbie AE, Parchman M, Tysinger J (2002). Guidelines for constructing a survey. Fam Med.

[12] Fast AM, Deibert CM, Hruby GW, Glassberg KI (2013). Evaluating the quality of Internet health resources in pediatric urology. J Pediatr Urol.

[13] Stinson JN, Tucker L, Huber A, Harris H, Lin C, Cohen L, Gill N, Lukas-Bretzler J, Proulx L, Prowten D (2009). Surfing for juvenile idiopathic arthritis: perspectives on quality and content of information on the Internet. J Rheumatol.

[14] Yeung TM, Mortensen NJ (2012). Assessment of the quality of patient-orientated Internet information on surgery for diverticular disease. Dis Colon Rectum.

[15] Hargrave DR, Hargrave UA, Bouffet E (2006). Quality of health information on the Internet in pediatric neuro-oncology. Neuro Oncol.

