# Supplementary material for: Are Parents Getting it Right? A Survey of Parents’ Internet Use for Children’s Health Care Information
Source: Interact J Med Res. 2015 Jun 22;4(2):e12. doi: 10.2196/ijmr.3790 (PMC4526953; doi:10.2196/ijmr.3790)
Supplement: Multimedia Appendix 1 [file ijmr_v4i2e12_app1.pdf]

Appendix

Which of the following websites are you familiar with?

Hospital/university based websites (eg. AboutKidsHealth)

Medical journal/reference websites (eg. Medscape,PubMed, eMedicine)

Public search engines (eg. Google,Bing, Yahoo)

Other health websites (MayoClinic, WebMD)

Popular parenting websites (Today's Parent)

Other

How frequently do you use the Internet/websites to search for information around your child's health?

Never

A few times a year)

Once a month

A few times a month

Every week

Every day

What type of device do you use to access children's health information on the Internet? (Please select all that apply)

Desktop computer

Laptop

Tablet device

Smartphone

None of the above

Other

What influences how often you use the internet/websites to search for information around your child's health

Which of the following do you go to FIRST when searching for information around your child's health?

Medline/Ovid

Medscape/PubMed/eMedicine

Web MD/MD Consult/Uptodate.com

Google/Bing/Yahoo/Ask.com

Wikipedia

AboutKidsHealth (SickKids)

Today's Parent

Other

Is there anyone you cross check information you find on websites with? Select from below

Family Physician

Pediatrician

Friends

Family

Friends/Family in healthcare

Spiritual Adviser

Other

Which of the following do you consider SAFE sources of information regarding your child's health?

Hospital/university based websites (eg. AboutKidsHealth)

Medical journal/reference websites (eg. Medscape,PubMed, eMedicine)

Government websites (ex. Ontario Ministry of Health and Long-Term Care, MedlinePlus, Health Canada

Child health specific websites run by healthcare professionals (ex. KidsHealth.org, HealthyChildren.org)

Public search engines (eg. Google,Bing, Yahoo)

Other health websites (MayoClinic, WebMD)

Popular parenting websites (Today's Parent)

Which of the following do you consider ACCURATE sources of information regarding your child's health?

Hospital/university based websites (eg. AboutKidsHealth)

Medical journal/reference websites (eg. Medscape,PubMed, eMedicine)
Government websites (ex. Ontario Ministry of Health and Long-Term Care, MedlinePlus, Health Canada

Child health specific websites run by healthcare professionals (ex. KidsHealth.org, HealthyChildren.org)

Public search engines (eg. Google,Bing, Yahoo)

Other health websites (MayoClinic, WebMD)

Popular parenting websites (Today's Parent)

Which of the following do you consider USEFUL sources of information regarding your child's health?

Hospital/university based websites (eg. AboutKidsHealth)

Medical journal/reference websites (eg. Medscape,PubMed, eMedicine)
Government websites (ex. Ontario Ministry of Health and Long-Term Care, MedlinePlus, Health Canada

Child health specific websites run by healthcare professionals (ex. KidsHealth.org, HealthyChildren.org)

Public search engines (eg. Google,Bing, Yahoo)

Other health websites (MayoClinic, WebMD)

Popular parenting websites (Today's Parent)

How likely are you to use each of the following websites in the next 12 months?

Hospital/university based websites (eg. AboutKidsHealth)

1 Unlikely
2
3
4
5
6
7 Likely

Medical journal/reference websites (eg. Medscape,PubMed, eMedicine)
Government websites (ex. Ontario Ministry of Health and Long-Term Care, MedlinePlus, Health Canada

1 Unlikely
2
3
4
5
6
7 Likely

Child health specific websites run by healthcare professionals (ex. KidsHealth.org, HealthyChildren.org)

1 Unlikely
2
3
4
5
6
7 Likely

Public search engines (eg. Google,Bing, Yahoo)

1 Unlikely
2
3
4
5
6

7 Likely

Other health websites (MayoClinic, WebMD)

1 Unlikely
2
3
4
5
6
7 Likely

Popular parenting websites (Today's Parent)

1 Unlikely
2
3
4
5
6
7 Likely

How likely are you to cross check information from the following websites with a trusted person?

Hospital/university based websites (eg. AboutKidsHealth)

1 Unlikely
2
3
4
5
6
7 Likely

Medical journal/reference websites (eg. Medscape,PubMed, eMedicine)
Government websites (ex. Ontario Ministry of Health and Long-Term Care, MedlinePlus, Health Canada

1 Unlikely
2
3
4

5
6
7 Likely

Child health specific websites run by healthcare professionals (ex. KidsHealth.org, HealthyChildren.org)

1 Unlikely
2
3
4
5
6
7 Likely

Public search engines (eg. Google,Bing, Yahoo)

1 Unlikely
2
3
4
5
6
7 Likely

Other health websites (MayoClinic, WebMD)

1 Unlikely
2
3
4
5
6
7 Likely

Popular parenting websites (Today's Parent)

1 Unlikely
2
3
4
5
6
7 Likely

Has your child ever been admitted to hospital before?

SickKids

Other hospitals (Trillium, Toronto East)

Emergency Room/Walk-in clinic

None of the above

Other

How many times has your child been admitted into hospital?

0

1-3

4-6

>6

What was the longest stay your child had during the admission?

<24 hours
1-5 days
A week
A few weeks
A month
A few months
Not applicable

Did you know SickKids has a dedicated Child health Website called AboutKids Health?

YES
NO

How likely are you to use AboutKids Health by SickKids? (move slider)

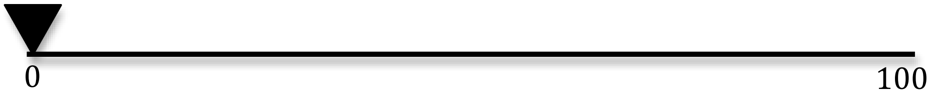

What devices are you likely to use to access AboutKids Health?

Desktop computer

Laptop

Tablet device

Smartphone

None of the above

What is your primary language?

How many boys do you have?

How many girls do you have?
